# Tuberculosis reactivation demonstrated by choroiditis and inflammatory choroidal neovascular membrane in a patient treated with immune checkpoint inhibitors for malignant mucosal melanoma

**DOI:** 10.1186/s12348-023-00374-x

**Published:** 2023-12-18

**Authors:** Melissa L. Murphy, Duncan Rogers

**Affiliations:** School of Medicine and Medical Sciences, Mater Misericordiae University Hospital, Eccles Street Dublin, 7, Dublin, Ireland

**Keywords:** Immune checkpoint inhibitors, Uveitis, Tuberculosis, Serpiginous-like Choroiditis, Choroidal Neovascular Membrane, Malignant Melanoma

## Abstract

**Purpose:**

To describe a complex case of ocular tuberculosis reactivation with anterior uveitis, choroiditis and inflammatory choroidal neovascular membrane (CNVM) following immune checkpoint inhibitor (ICPI) treatment of malignant mucosal melanoma.

**Methods:**

A retrospective collection of medical history, clinical findings and multimodal imaging with literature review of the topic was conducted.

**Results:**

A 52-year-old Romanian female developed reduced vision and photophobia after three cycles of ICPI therapy comprised of ipilimumab and nivolumab. Bilateral anterior uveitis, multiple left eye choroidal lesions and a CNVM were confirmed using slit-lamp examination with ancillary multimodal imaging. Retinal changes in the right eye as well as a history of previously treated posterior uveitis and high-risk ethnicity increased clinical suspicion for ocular tuberculosis (TB) reactivation. The diagnosis was confirmed by TB positivity on polymerase chain reaction (PCR) analysis of lung aspirate followed by significant clinical improvement on systemic anti-tubercular therapy (ATT), systemic steroids and anti-vascular endothelial growth factor (VEGF) therapy.

**Conclusions:**

ICPIs can cause a myriad of ocular issues, both by primary immunomodulatory effects as well as secondary reactivation of latent disease.

## Introduction

Ipilimumab and nivolumab are commonly used immune checkpoint inhibitors (ICPIs) that induce activation of T-cells against cancer cells, therefore blocking the mechanisms cancer cells use to disguise themselves as regular human body components [1]. These novel drugs have proved invaluable in treating previously unresectable metastatic cancer, primarily melanoma, significantly prolonging life expectancy [2]. Immune-related adverse events (IRAEs) are a commonly associated side effect of ICPIs and can cause visual disturbance [3, 4]. In this brief report we describe an unusual case of bilateral uveitis, caused primarily by reactivation of latent ocular TB, characterised by multiple choroidal lesions with secondary inflammatory CNVM.

## Case presentation

**Fig. 1:**
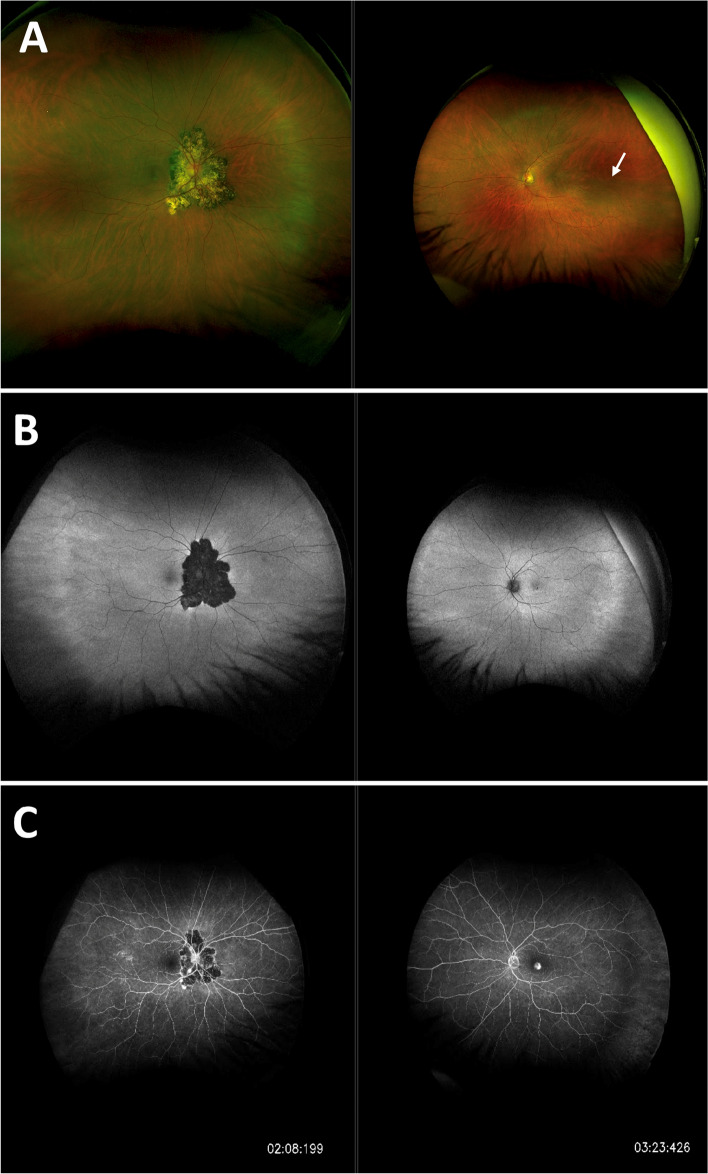
**A:** Optos ultra-widefield pseudocolour fundus image of both eyes showing mottled chorioretinal circumpapillary pigmentation in the right eye and macular haemorrhage in the left eye (white arrow). **B:** Fundus auto-fluorescence (FAF) of both eyes on initial presentation. **C:** Optos ultra-widefield fluoroscein angiography showing areas of mottled hyper- and hypofluorescence in the right eye and a central macular area of leakage in the left eye (late shots)

A 52-year-old female presented to the Eye Casualty complaining of a 4-week history of blurred vision in her left eye and a 2-week history of bilateral photosensitivity. On examination, best corrected visual acuity (BCVA) was 6/12 in the right eye and 6/24 in the left eye. Corneal exam was normal with no keratic precipitates. There was bilateral anterior uveitis with + 2 cells in the right eye and + 0.5 cells in the left eye. Intra-ocular pressure (IOP) was raised in the right eye at 40 mmHg and borderline at 19 mmHg in the left eye. There was no vitritis. Dilated fundoscopy revealed a large mottled area of chorioretinal pigmentation in the right eye centred around the optic nerve and a left macular haemorrhagic lesion suspicious for a CNVM (Fig. 1A).

She reported previous treatment in Romania (a high TB burden country, with the highest rates of TB in the European Union [5]) several years earlier of an infective condition affecting her right retina requiring systemic antibiotic and steroid therapy for a 6-month period. No further details were available and she had no other ophthalmic history. There were no self-reported ophthalmic issues since treatment completion and she stated her baseline visual acuity prior to this presentation was normal in both eyes. She had a significant medical history of malignant mucosal melanoma diagnosed six months prior to this ophthalmic presentation. Biopsy confirmed a primary vaginal melanoma, nodular histological subtype, Clarke’s level 3, mitotic index 5/mm^2^, Breslow thickness 12mm with extensive ulceration and no adjacent lympho-vascular or perineural invasion. A staging positron emission tomography-computed tomography (PET-CT) performed at the time of diagnosis revealed a 3.8cm fluorodeoxyglucose (FDG) avid vaginal mass with a standardised uptake value (SUV) of 5.9 suggesting a malignant lesion. A complete vaginectomy was performed followed by pelvic radiation. Deep margins were positive on histology and the decision was made to initiate adjuvant immunotherapy with ipilimumab and nivolumab. She had completed her third cycle 6 days prior to this presentation. She had no other medical history apart from mild hypertension not requiring medication. She was a non-smoker with no alcohol or illicit drug use. She was of Romanian ethnicity but had been living in Ireland for many years. She reported travelling to Romania occasionally during her time living in Ireland to visit relatives but no known TB contacts. She worked in a restaurant and was married with one son.

**Fig. 2:**
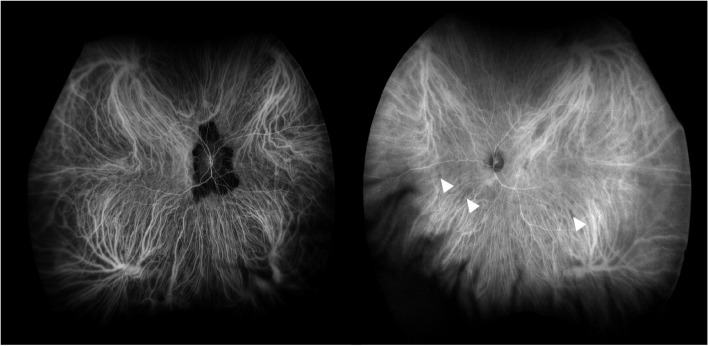
Indocyanine green angiography (ICGA) images with multiple hypofluorescent dark dots (white triangles) in the left eye (late shots)

**Fig. 3:**
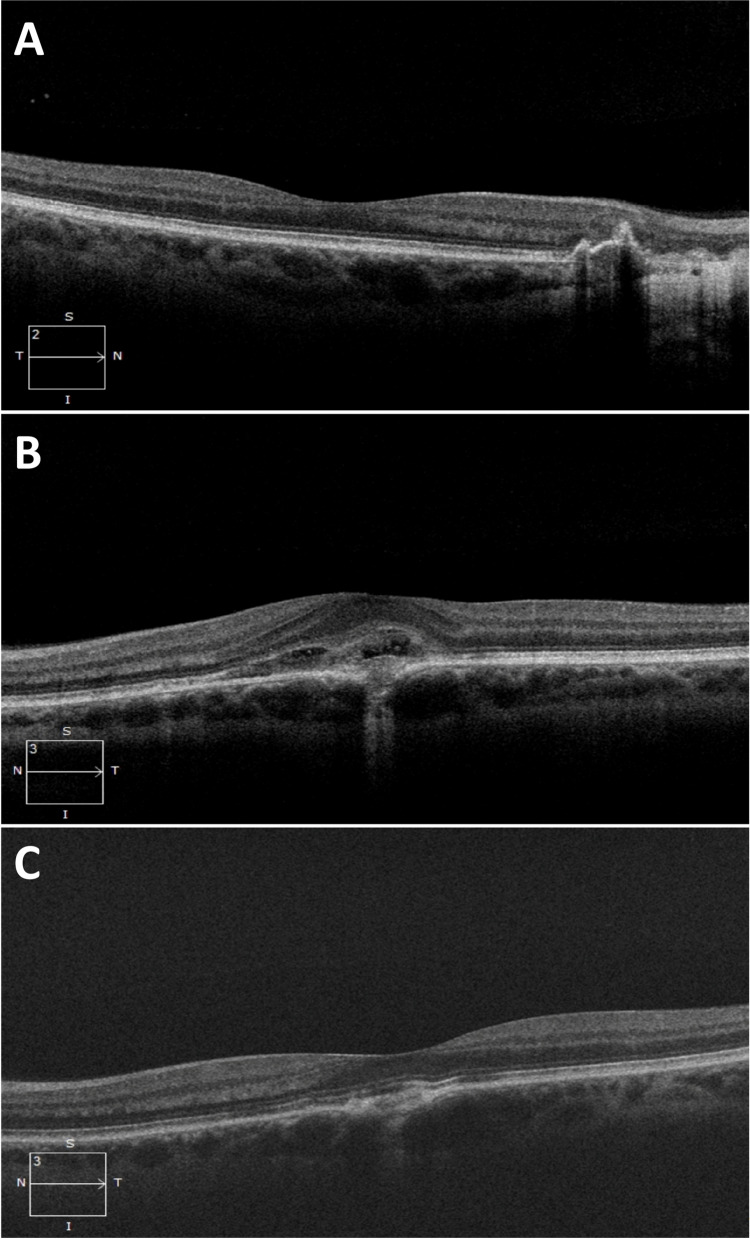
**A:** Optical coherence tomography (OCT) image of right macula at presentation showing peripapillary disruption of all outer retinal layers with no associated fluid. **B:** OCT image of left macula at presentation showing intraretinal and subretinal foveal fluid with loss of normal foveal contour. **C:** OCT image of left macula six weeks post second intravitreal Bevacizumab showing resolution of fluid

Fundus autofluorescence (FAF) revealed the right retinal lesion to be inactive in appearance with no hyper-autofluorescence (Fig. 1B). Fundus fluorescein angiogram (FFA) showed a subfoveal area of leakage in the left eye compatible with an active CNVM (Fig. 1C). There was no evidence of vasculitis. Indocyanine green angiography (ICGA) revealed multiple hypofluorescent spots in the left eye (Fig. 2). No choroidal granulomas were visualised. Optical coherence tomography (OCT) images of her right eye showed peripapillary disruption of the outer retinal layers without fluid or secondary macular disturbance (Fig. 3A). OCT images of her left eye showed central intraretinal and subretinal fluid with loss of the normal foveal contour (Fig. 3B). She underwent a full uveitis blood investigation panel including *Treponema Pallidum*, *Toxoplasma gondii*, *Toxocara canis*, Herpes Simplex Virus, Varicella Zoster Virus, Cytomegalovirus and other autoimmune inflammatory conditions associated with posterior uveitis which were all negative. Aqueous and vitreous sampling was not performed.

Due to the quiescent serpiginous-like choroiditis (SLC) appearance of the right fundus, previously reported systemic treatment of a right eye infectious condition, growing up in a country with endemic TB, the presence of multiple choroidal lesions and an active CNVM, reactivation of latent TB in her left eye was suspected. She underwent a left eye intravitreal anti-VEGF Bevacizumab injection on the day of her presentation to target the CNVM. She was given a stat dose of Acetazolamide 250mg and was initiated on G Brinzolamide/Timolol and G Apraclonidine to her right eye to reduce IOP.

On discussion with her oncologist her fourth cycle of immunotherapy was postponed due to the development of ocular IRAEs and she was initiated on a tapering systemic regime of Prednisolone 1mg/kg over 8 weeks by the treating oncologist, on the assumption that this was likely an ICPI-induced uveitis. This was in conjunction with a topical steroid taper of G Pred Forte over a 6-week period to target the anterior chamber activity. Mediastinoscopy revealed a non-caseating granulomatous lymphadenitis, possible aetiologies including TB and sarcoidosis. Ancillary tests including Ziehl–Neelsen stain was negative, washout Auramine stain was negative and TB culture was negative. PCR testing with the GeneXpert MTB/RIF assay was positive for TB on endobronchial washout. A QuantiFERON-TB Gold blood test had been performed prior to ICPI initiation and again following this ophthalmic presentation and was reported as negative in both instances. It was deemed to have reduced sensitivity due to prior systemic immunosuppression with ICPI. On consultation with infectious disease specialists, she was initiated on systemic ATT within 1 week of her ocular presentation using rifampicin, isoniazid, pyrazinamide and moxifloxacin to cover for potential multi-drug-resistant tuberculosis given her ethnic background.

**Fig. 4:**
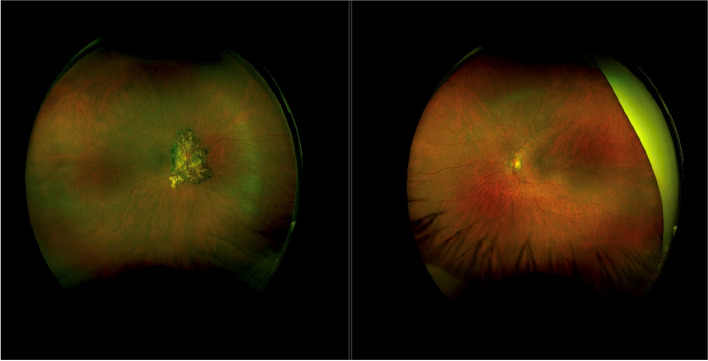
Optos wide-field pseudocolour image of both eyes following completion of anti-VEGF and systemic anti-TB therapy with resolution of left macular haemorrhage

She underwent a second left eye intravitreal Bevacizumab 2-months later with an excellent response. On all subsequent eye examinations BCVA was maintained at 6/5 in both eyes, OCT remained dry (Fig. 3C) and she had no evidence of uveitis. Her right fundal lesion remained unchanged (Fig. 4). She was recommenced on a fourth cycle of immunotherapy two months after the development of her uveitis without ocular sequelae.

Unfortunately, a repeat staging PET-CT scan performed three months after her presentation to the Eye Casualty revealed disseminated metastatic disease. The ATT was tolerated well initially, however, four months into treatment she developed jaundice with grossly deranged liver function tests (ALT 1569, GGT 105, ALP 126, bilirubin 70). A liver ultrasound confirmed multiple large liver metastasis with central necrosis. ATT was discontinued prematurely due to the potential risk of fulminant hepatitis with isoniazid. She was discharged to community palliative care and passed away within one year of her diagnosis.

## Discussion

ICPIs are classified according to the immune ligand targeted. Ipilimumab is a monoclonal antibody directed against cytotoxic T-lymphocyte antigen-4 (CTLA-4) and nivolumab targets programmed cell death protein 1 (PD-1) [1]. A 2022 randomised clinical trial comparing ipilimumab and nivolumab combination therapy versus monotherapy in untreated unresectable stage III or stage IV melanoma reported the longest median overall survival (OS) reported to date: 57% OS at 6.5 years with *BRAF*-mutant tumours and 46% OS at 6.5 years in *BRAF*-wild type tumours [2]. While these treatments have been proven highly successful in treating cancers such as metastatic melanoma, lung and colon cancer, they characteristically induce side-effects known as IRAEs which can affect the eye [3, 4]. A recent large meta-analyses reported diarrhoea, colitis, rash, pruritus, hypophysitis, hypothyroidism, hyperthyroidism and pneumonitis as the most frequent IRAEs, with each ICPI and cancer type associated with a different toxicity profile [3]. The incidence of all-grade uveitis was 2.0% with ipilimumab and nivolumab combination therapy compared to < 1% for ipilimumab, nivolumab and pembrolizumab monotherapies [3].

In addition to uveitis, potential ophthalmic IRAEs reported include orbital inflammation/ocular myositis [3, 6, 7], peripheral ulcerative keratitis [3], retinal vasculitis [8], melanoma associated retinopathy (MAR) like retinopathy [9], Vogt-Koyanagi Harada like posterior uveitis [10, 11], cranial neuropathies [7, 12], optic neuritis [7] and ocular myasthenia [7, 13]. Nivolumab is associated with the highest rate of ocular IRAEs overall whilst ipilimumab has the strongest association with uveitis [13]. Cases are managed on an individual basis with a multidisciplinary team (MDT) approach. Intraocular or oral corticosteroids can be initiated to reduce inflammation and occasionally therapeutic plasma exchange has been utilised in refractory cases [14]. Temporary or permanent ICPI cessation may be necessary [7, 14].

In this case, the patient presented with reduced vision and photophobia following completion of her third round of ICPIs for malignant vaginal melanoma. The diagnostic challenge in this case was to identify the primary aetiology for the uveitis which comprised of a bilateral non-granulomatous anterior uveitis, right eye chorioretinal changes and an active left eye CNVM with scattered choroidal lesions. The clinical appearance of her right fundus was suggestive of a quiescent previously treated SLC. The left eye had multiple choroidal lesions evident on ICGA imaging with a secondary inflammatory CNVM. This raised the index of suspicion for a TB related uveitis, potentially caused by ICPI induced reactivation of latent TB, which has been reported [15]. SLC is an immune-mediated inflammation of the choriocapillaris caused by *Mycobacterium tuberculosis* (Mtb) growth within the retinal pigment epithelium (RPE) [16]. Tubercular SLC can be difficult to manage due to poor RPE penetration with ATT and the requirement for prolonged immunosuppression to dampen the associated inflammatory response [16]. Fundal findings and FFA imaging did not show activity in this lesion which was likely treated a number of years prior to this presentation with prolonged systemic medication.

The Collaborative Ocular Tuberculosis Study published in 2020 provides guidance on the management of ocular TB with expert consensus to initiate ATT in tubercular choroiditis in the presence of a positive immunologic test plus radiologic features suggestive of TB [17]. For tubercular SLC and tuberculoma, positive results from one immunologic test alone is considered sufficient to initiate ATT [17]. In the left eye tubercular choroiditis as evidenced by the ICG findings with an inflammatory CNVM was the most likely aetiology for this posterior uveitis.

ICPI-induced enhancement of effector T cells with associated excessive tumour necrosis factor-alpha (TNF- α) secretion is hypothesised to cause destruction of granuloma extracellular matrix in latent TB with enhancement of Mtb growth and spread [15]. Lung histology showed a granulomatous lymphadenitis with TB confirmed on GeneXpert MTB/RIF assay. Interestingly, QuantiFERON-TB Gold blood tests performed both prior to ICPI commencement and following this presentation were negative. This finding is reflected in a large meta-analysis of interferon-gamma release assays (IGRAs) used in the immune-diagnosis of TB, which found up to 20% of active TB patients having a negative TB blood test [18]. ICGA revealed multiple round hypofluorescent dark dots which have been reported in up to 100% of eyes with presumed intraocular TB [19]. Treatment was centred around temporary discontinuation of ICPIs, topical steroids, systemic ATT and systemic steroids. Anti-VEGF therapy was employed successfully to treat the active inflammatory CNVM secondary to choroiditis in the left eye and BCVA was excellent post treatment. Following this, ICPIs cycle four was recommenced but unfortunately due to the advanced stage of disease, treatment was unsuccessful and the patient passed away. This case highlights the importance of the multiple ways in which ICPIs can cause ocular complications and the importance of an MDT approach to complex medical cases.

## Conclusion

An unusual case of bilateral anterior non-granulomatous uveitis with raised IOP, right eye chorioretinal scarring and left eye choroidal lesions with active CNVM highlights the multiple complex ways ICPIs can alter immunoreactivity causing visual problems.

## Data Availability

Data sharing is not applicable to this article as no datasets were generated or analysed from this case study.

## Abbreviations

CNVM: Choroidal neovascular membrane
ICPIs: Immune checkpoint inhibitors
TB: Tuberculosis
PCR: Polymerase chain reaction
ATT: Anti-tubercular therapy
anti-VEGF: Anti-vascular endothelial growth factor
IRAEs: Immune-related adverse events
BCVA: Best corrected visual acuity
IOP: Intraocular pressure
PET-CT: Positron emission tomography-computed tomography
FDG: Fluorodeoxyglucose
SUV: Standardised uptake value
FAF: Fundus autofluorescence
FFA: Fundus fluorescein angiogram
ICGA: Indocyanine green angiography
OCT: Optical coherence tomography
SLC: Serpiginous-like choroiditis
CRLA-4: Cytotoxic T-lymphocyte antigen-4
PD-1: Programmed cell death protein 1
OS: Overall survival
MDT: Multidisciplinary team
Mtb: Mycobacterium tuberculosis
RPE: Retinal pigment epithelium
TNF- α: Tumour necrosis factor-alpha
IGRAs: Interferon-gamma release assays
